# Effects of a single-cell protein source from *Paecilomyces* var*iotii* on diet digestibility and palatability and intestinal functionality of adult dogs

**DOI:** 10.3389/fvets.2026.1787800

**Published:** 2026-06-10

**Authors:** Renata Bacila Morais dos Santos de Souza, Eduarda Lorena Fernandes, Lorenna Nicole Araújo Santos, Laiane da Silva Lima, Heloísa Lara Silva, Simone Gisele de Oliveira, Ananda Portella Félix

**Affiliations:** Research Laboratory in Canine Nutrition, Department of Animal Sciences, Federal University of Paraná, Curitiba, Brazil

**Keywords:** beta-glucans, fermentative metabolites, filamentous fungi, microbiota, sustainable ingredients

## Abstract

**Introduction:**

This study aimed to evaluate the effects of a single-cell protein (SCP) source from *Paecilomyces variotii* on the apparent total tract digestibility (ATTD) of macronutrients and energy, diet palatability, fecal fermentative metabolites, and microbiota in dogs.

**Materials and methods:**

Five extruded diets containing 0, 4, 8, 12, and 16% of SCP were manufactured. To isolate the metabolizable energy (ME) and the ATTD of the SCP, an additional test diet was manufactured containing 80% of the 0% diet and 20% of SCP. In Experiment I, 15 adult Beagle dogs were distributed in a randomized block design with 5 diets (0 to 16% SCP) and two periods of 21 days each, totaling 6 repetitions/treatment. In Experiment II, for the palatability test, 16 adult dogs were used, comparing the diets: 0 vs. 4% SCP; 0% vs. 8% SCP; and 4% vs. 16% SCP. In Experiment III, the SCP digestibility was estimated by the substitution method with 12 adult Beagle dogs.

**Results:**

The SCP presented ATTD of dry matter (DM) = 64.3%; ATTD of crude protein = 83.9%; ATTD of acid-hydrolyzed ether extract = 78.3%; and ME of 3843.3 kcal/kg. The ATTD of DM, organic matter, gross energy and the ME of the diets decreased linearly as the dietary inclusion of SCP increased (0 to 16% SCP; *p* < 0.05). There was a quadratic effect in fecal concentrations of propionate, butyrate, and total short-chain fatty acids, and a linear increase in isobutyrate and total branched-chain fatty acids, with the dietary inclusion of SCP (*p* < 0.05). Animals fed the 8% SCP diet presented an increase in alpha-diversity indexes (*p* < 0.05). Dogs fed the 4% SCP diet presented higher fecal abundance of *Lactobacillus* and *Limosilactobacillus*, when compared to the 0% group (*p* < 0.05). Besides, a higher fecal abundance of *Lactobacillus* and *Butyricicoccus* and lower abundance of *Enterococcus*, and *Enterocloster* was observed in dogs fed the 8% SCP diet compared to the 0% group (*p* < 0.05).

**Conclusion:**

These results demonstrate that the dietary inclusion of 4 and 8% SCP promotes less impact on diet digestibility and may beneficially modulate the fecal microbiome and its metabolites in dogs, without affecting diet palatability.

## 1 Introduction

The growing global demand for protein has driven the pet food industry to seek alternative ingredients with a lower environmental impact, including both novel protein sources, agro-industrial by-products and underutilized food streams (1). Research has therefore focused on innovative protein sources for dogs, with particular attention to microorganisms such as fungi and yeasts, which are notable for their high protein content, available both as whole biomass and in concentrated forms. These ingredients are commonly referred to as “microbial ingredients” or “single-cell protein” (SCP) (2).

The use of SCP into pet food represents a promising option from nutritional, economic, and sustainability perspectives. Its production is independent of seasonal factors, present high substrate conversion efficiency, and rapid growth, which together ensure elevated productivity (3). In addition, SCP provides high-value nutrients while reducing reliance on arable land, fresh water, and climate-sensitive resources, thereby contributing to a lower carbon footprint compared with conventional protein sources (4, 5).

In this context, *Paecilomyces* var*iotii* emerges as a promising SCP source. This versatile filamentous fungus thrives on a wide range of low-cost, organic-rich side streams, including by-products from bioethanol production as well as agricultural and lignocellulosic residues (6, 7). The SCP from *Paecilomyces* var*iotti* typically contains around 600 g/kg crude protein on a dry matter basis and has a low-fat content (less than 40 g/kg) (2, 5). It also contains essential amino acids such as threonine, lysine, and methionine (8). In addition, it contains *β*-glucans, present both within the cell walls (9) and as secreted extracellular polysaccharides (10). These compounds may confer additional health benefits for dogs through their immunomodulatory properties and their potential to influence gut microbiota composition and fermentative metabolites production (11, 12).

Early studies conducted in the 1970s and 1980s demonstrated the safety and nutritional viability of *Paecilomyces* var*iotii* SCP in pigs and laying hens (13–15). More recently, its use in aquaculture has been investigated, not only as a protein source but also for its functional effects on the fecal microbiota and immune response of fish (16). However, no studies to date have evaluated the nutritional or functional impacts of *P.* var*iotii* SCP in dogs. We hypothesized that the dietary inclusion of *P. variotii* SCP would influence nutrient digestibility and positively modulate fecal microbiota and fermentative metabolites, without adversely affecting diet palatability in dogs. Accordingly, the aim of this study was to assess the effects of *P.* var*iotii* on macronutrient and energy digestibility, diet palatability, and fecal microbiota and its metabolites in dogs. Additionally, we aimed to isolate the SCP digestibility in dogs.

## 2 Materials and methods

### 2.1 Experiment I: digestibility, fecal characteristics, fermentative metabolites, and microbiota

#### 2.1.1 Animals and facilities

The Ethics Committee on Animal Use of the Agrarian Sciences Sector of the Federal University of Paraná, Curitiba, PR, Brazil, approved the use of animals for this study under protocol n. 044/2023. Fifteen adult Beagle dogs (7 males and 8 females), 1 year of age, neutered/spayed, and with an average body weight of 10.43 ± 1.04 kg were used. The dogs had a mean body condition score (BCS) of 4.5 ± 0.51, assessed on a scale of 1 to 9, according to Laflamme (17). The animals were submitted to clinical examination before and after the experimental period and were healthy.

The dogs were individually housed in brickwork kennels (5 m long x 2 m wide), containing a bed and free access to fresh water. During most of the experimental period, the dogs had access to an outdoor area of 1,138 m^2^ for 4 h/day for voluntary exercise and socialization. During the feces collection period, the dogs were individually housed in kennels. The facilities had side wall bars that allowed visual and limited interaction with neighboring dogs. Besides, the animals received extra attention and environmental enrichment inside the kennel during this period. The temperature ranged from 16 °C to 28 °C, with a 12 h light–dark cycle (light from 6:00 a.m. to 6:00 p.m.). The dogs were supervised by the researchers and the veterinarian responsible for the laboratory throughout the experimental period. After the experimental period, all dogs remained housed in the institutional experimental kennel facility, where they continue to be maintained and monitored according to institutional care protocols.

#### 2.1.2 Ingredients, experimental diets, and design

A total of five diets were evaluated: 0 (control), 4, 8, 12, and 16% inclusion of SCP (*Paecilomyces* var*iotii* KCL-24, Pekilo® mycoprotein, Enifer, Espoo, Finland). The diets were formulated to be isoproteic and isoenergetic and to meet the nutritional requirements of adult dogs according to FEDIAF (18). The ingredients and chemical composition of the experimental diets and of the SCP are presented in Tables 1–3, respectively. The mycotoxin profile of the SCP, provided by the manufacturer, has been included in Table 3 to demonstrate the ingredient’s safety.

**Table 1:** Ingredients of experimental diets.

Ingredients (g/kg)	Single cell protein (%)					
	0	4	8	12	16	20^5^
Corn	303.58	301.62	296.02	287.25	278.47	242.86
Poultry by-product meal	273.85	236.33	199.24	162.51	125.78	219.08
Broken rice	200.00	200.00	200.00	200.00	200.00	160.00
Soybean meal^1^	100.00	100.00	100.00	100.00	100.00	80.00
Poultry fat	55.26	58.72	62.21	65.75	69.28	44.21
Liquid palatant	20.00	20.00	20.00	20.00	20.00	16.00
Common salt	8.00	8.00	8.00	8.00	8.00	6.40
Cellulose	7.02	2.79	0.00	0.00	0.00	5.62
Phosphoric acid	7.00	7.00	7.00	7.00	7.00	5.60
Mineral–vitamin supplement^2^	6.40	5.93	5.46	4.99	4.52	5.12
Potassium chloride	6.21	5.16	4.13	3.12	2.10	4.97
Choline chloride	4.69	4.74	4.79	4.83	4.88	3.75
Dl-methionine	4.00	4.00	4.00	4.00	4.00	3.20
Antifungic	1.50	1.50	1.50	1.50	1.50	1.20
Taurine	1.50	1.50	1.50	1.50	1.50	1.20
BHA/BHT^3^	1.00	1.00	1.00	1.00	1.00	0.80
Calcareous	0.00	1.70	5.14	8.56	11.97	0.0
*Paecilomyces variotii* ^4^	0.00	40.00	80.00	120.00	160.00	200.00

1 450 g crude protein/kg.

2 Enrichment per kg of product: vitamin A (retinol), 20,000 IU; vitamin D3, 2,000 IU; vitamin E (alpha-tocopherol), 48 mg; vitamin K3, 48 mg; vitamin B1, 4 mg; vitamin B2, 32 mg; pantothenic acid, 16 mg; niacin, 56 mg; choline, 800 mg; Zn as zinc oxide, 150 mg; Fe as ferrous sulfate, 100 mg; Cu as copper sulfate, 15 mg; I as potassium iodide, 1.5 mg; Mn as manganese oxide, 30 mg; Se as sodium selenite, 0.2 mg; antioxidant, 240 mg.

3 BHT, Butylated Hydroxytoluene.; BHA, 3-tert-Butyl-4-hydroxyanisole.

4 P. variotii (PEKILO® mycoprotein, Enifer, Espoo, Finland).

5 Ingredients of the test diet used for the substitution method.

**Table 2:** Analyzed chemical composition (g/kg of dry matter) of experimental diets.

Item	Single cell protein (%)					
	0	4	8	12	16	20^1^
Dry matter	94.67	93.72	94.87	94.20	94.44	92.00
Crude protein	28.65	28.56	28.15	28.14	28.40	33.00
Ether extract in acid hydrolysis	13.71	14.17	14.66	14.11	13.66	11.85
Crude fiber	2.26	1.91	2.23	2.10	2.09	5.17
Ash	5.97	5.80	5.72	5.60	5.62	5.38
Calcium	0.96	0.84	0.82	0.84	0.91	0.69
Phosphorus	0.72	0.75	0.74	0.66	0.76	0.93
Gross energy (kcal/kg)	5019.37	5016.94	5003.79	5013.54	5009.00	5103.35

1 Analyzed chemical composition of the diet used for the substitution method.

**Table 3:** Analyzed chemical composition of *P. variotti* (SCP).

Item (g/kg, dry matter)^1^	SCP
Dry matter	880.50
Crude protein	657.50
Ether extract	37.20
Total dietary fiber	229.40
Soluble fiber	14.40
Insoluble fiber	215.0
Ash	75.00
Calcium	0.40
Phosphorous	16.20
Amino acids (g/kg, dry matter)^1^	
Aspartic acid	47.60
Glutamic acid	60.26
Serine	25.60
Glycine	28.35
Histidine	11.82
Arginine	39.10
Threonine	25.18
Alanine	34.06
Proline	20.11
Tyrosine	17.76
Valine	26.33
Methionine	8.69
Cystine	4.11
Isoleucine	21.97
Leucine	38.31
Phenylalanine	21.79
Lysine	39.25
Total aminoacids sum	470.25
Mycotoxin (mcg/kg, dry matter)^1^	
Total aflatoxins (B1 + B2 + G1 + G2)	<2.00 (LQ^2^)
Fumonisins (B1 + B2)	520.00
Deoxynivalenol	150.00
Ochratoxin	2.01
Trichothecenes (T-2)	<25.00 (LQ^2^)
Zearalenone	<20.00 (LQ^2^)

1 Manufacturer’s data (Enifer, Espoo, Finland).

2 LQ, Limit of quantification.

The experimental diets were processed at the Extrusion Laboratory of the College of Agrarian and Veterinarian Sciences, São Paulo State University (UNESP), Jaboticabal, SP, Brazil. A single lot of raw materials was used for all diets. Ingredients were weighed and mixed before being ground in a hammer mill (Tigre, Moinhos Tigre, São Paulo, SP), fitted with a 0.8 mm sieve, and extruded in a single-screw extruder (Model Mex-250, Manzoni Industria Ltda, Campinas, SP), with an average extrusion capacity of 250 kg/h. The temperature of the extruder preconditioner was kept higher than 85 °C by direct steam injection. After extrusion, the kibbles were dried in a forced air dryer at 105 °C for approximately 20 min and coated with poultry fat and liquid palatant.

The animals were fed twice a day (08:00 a.m. and 04:00 p.m.) in amounts sufficient to supply the metabolizable energy (ME) requirement of adult dogs in maintenance as recommended by the National Research Council (NRC) (19). Water was provided *ad libtum.*

The experiment followed a randomized blocks design, with two blocks (periods) of 21 days each. Each 3 dogs were fed one of the diets per period, totaling 6 repetitions per treatment at the end of the 2 periods.

#### 2.1.3 Digestibility assay and fecal characteristics

The digestibility trial followed the total fecal collection method recommended by the Association of American Feed Control Officials (AAFCO) (20), with 5 days of total fecal collection (days 17 to 21 and 38 to 42). Feces were collected at least twice a day, weighed, stored in individual plastic bags previously identified, covered, and stored in a freezer at −14 °C to be analyzed later. At the end of the collection period, the feces were thawed at room temperature and homogenized separately, forming a composite sample of each animal.

Feces were dried in a forced ventilation oven (320-SE, Fanem, São Paulo, Brazil) at 55 °C for 72 h or until reaching a constant weight. After drying, the feces and the experimental diet were ground using a 1 mm sieve in a grinder (Arthur H. Thomas Co., Philadelphia, PA, United States) and analyzed for dry matter (DM) at 105 °C for 12 h, nitrogen (N, method 954.01), and crude protein (CP), which was calculated as N × 6.25, crude fiber (method 994.13), ash (method 942.05), acid-hydrolyzed ether extract (AEE; method 942.05), calcium (method 972.02), and phosphorus content (method 965.17). All analyses followed the recommendations of the Association of Official Analytical Chemists (AOAC) (21). Gross energy (GE) was determined in a calorimeter pump (IKA C2000 Basic, IKA-Werke, Staufen, Germany).

Fecal characteristics were evaluated during the collection period by total dry matter (DMf) content and fecal consistency by score. An aliquot of feces (2 g) was dried at 105 °C for 48 h to determine DMf. Fecal score was always evaluated by the same researcher, who assigned points from 1 to 5, being: 1 = feces are soft and have no defined shape; 2 = feces are soft and poorly formed; 3 = feces are soft, formed, and moist; 4 = feces are well-formed and consistent; and 5 = feces are well-formed, hard and dry (22).

Fecal pH was analyzed in feces collected up to 15 min after spontaneous defecation on days 21 and 42 of the experiment and was measured using a digital pH meter (331, Politeste Instrumentos de Teste Ltda., São Paulo, SP, Brazil). For this, 3 g of fresh feces were diluted in 30 mL of distilled water.

#### 2.1.4 Fecal fermentative metabolites and microbiota

Fresh stool samples (up to 15 min after defecation) were collected (one sample per dog) and immediately homogenized for fermentative metabolites and microbiota analysis on days 21 and 42 of the experiment. Fecal ammonia concentrations were determined according to de Brito et al. (23). Briefly, 5 g of fresh feces were incubated in a 500 mL lidded glass balloon, containing 250 mL distilled water, for 1 h. Then, three drops of octyl alcohol (1-octanol) and 2 g of magnesium oxide were added to the solution, subsequently distilled in a Macro-Kjeldahl apparatus, and recovered in a beaker containing 50 mL boric acid. Finally, ammonia was titrated using standardized sulfuric acid at 0.1 N.

For determination of short-chain (SCFA, acetate, butyrate, propionate, and valerate), branched-chain (BCFA, isovalerate, isobutyrate and 4-methyl valerate), and other volatile fatty acids (hexanoic and heptanoic), fresh feces were collected up to 15 min after defecation. In a plastic container with a lid, 10 g of stool sample was weighed and mixed with 30 mL of 16% formic acid. This mixture was homogenized and stored in a refrigerator at 4 °C for a period of 3–5 days. After this period the solutions were centrifuged at 2500 gx (2 K15, Sigma, Osterode am Hans, NI, Germany) for 15 min. At the end of centrifugation, the supernatant was separated and subjected to further centrifugation. Each sample underwent three centrifugations, and at the end of the last one, part of the supernatant was transferred to a properly labeled eppendorf tube for subsequent freezing at – 14 °C. Afterward, the samples were thawed and underwent new centrifugation at 18000 gx for 15 min (Rotanta 460 Robotic, Hettich, Tuttlingen, BW, German). Both centrifugations were conducted under refrigeration (approximately 5 °C). Fecal SCFA, BCFA and other volatiles were analyzed by gas chromatography (Shimadzu, model GC-2014, Kyoto, Honshu, Japan), using a glass column (Agilent Technologies, HP INNO wax—19,091 N, Santa Clara, CA, United States of America) 30 m long and 0.32 mm wide. The injected volume of the supernatant was set to 1 μL. Nitrogen was used as the carrier gas with a flow rate of 3.18 mL/min. The working temperatures were 200 °C at the injector, 240 °C at the column (at a speed of 20 °C/min), and 250 °C at the flame ionization detector.

For evaluation of the fecal microbiota, approximately 2 g of sample was taken from the interior of the freshly collected stool of the animals that consumed the 0, 4 and 8% diets. The feces were placed in a sterile Eppendorf tube and stored in a − 80 °C freezer until the moment of the analysis. For DNA extraction from the samples, the commercial kit “ZR Fecal DNA MiniPrep®” from Zymo Research (Zymo Research, Irvine, CA, United States) was used following the protocol recommended by the manufacturer.

The extracted DNA was quantified by spectrophotometry at 260 nm using the NanoDrop® 2000 spectrophotometer (Thermo Scientific, Wilmington, VA, United States). To evaluate the integrity of the extracted DNA, all samples were run by electrophoresis in a 1% agarose gel, stained with a 1% ethidium bromide solution, and visualized with ultraviolet light in a transilluminator. A 460-base segment of the V4 hypervariable region of the 16S rRNA gene was amplified using the universal primers 515F and 806R and the following polymerase chain reaction (PCR) conditions: 94 °C for 3 min; 18 cycles of 94 °C for 45 s, 50 °C for 30s, and 68 °C for 60s; followed by 72 °C for 10 min. From these amplifications, a metagenomic library was built using the commercial Nextera DNA Library Preparation Kit from Illumina® (San Diego, CA, United States). The amplifications were pooled and afterward sequenced in the Illumina® “MiSeq” sequencer (24). The obtained reads were analyzed on the Quantitative Insights into Microbial Ecology (QIIME) platform (25, 26), followed by a workflow of low-quality sequences removal, filtration, chimera removal, and taxonomic classification. The sequences were classified into bacterial genera by recognizing amplicon sequence variants (ASVs), in this case, the homology of the sequences when compared against a database. To compare the sequences, the GTDB 202 update for 2021 of the Genome Taxonomy Database ribosomal sequence database was used (27). In order to normalize the data and not compare samples with different numbers of reads, 368,648 reads per sample were used to generate the classification of bacterial communities by ASV identification.

#### 2.1.5 Calculations and statistical analysis

The organic matter (OM) was calculated by:


OM(%)=100−Ash(%)

Based on the laboratory results, the coefficients of total tract apparent digestibility (ATTD) and metabolizable energy (ME) were calculated according to AFFCO (20):


$$\text{ATTD}\;(\%)=\frac{\text{nutrient intake}\;(g)-\text{nutrient excretion}\;(g)}{\text{nutrient intake}\;(g)}x\;100$$

$$\mathrm{ME}\;(\frac{\text{kcal}}{\mathrm{kg}})=\frac{\left\{ \begin{array}{l} {}[\mathrm{GE}\;\text{intake}\;(g)-\mathrm{GE}\;\text{excretion}\;]-\\ {}[\mathrm{CP}\;\text{intake}\;(g)-\mathrm{CP}\;\text{excretion}\;(g)]\;x\;1.25 \end{array}\}\;\right.}{\text{feed intake}\;(g)}$$

The ATTD, ME, DM intake, fecal characteristics, fermentative metabolites, and alpha-diversity indexes data were submitted to the Shapiro–Wilk normality test. A linear mixed model was applied to analyze the data, considering the dietary inclusion of SCP as a fixed effect and periods and animals as random effects. Linear and quadratic effects were tested (*p* < 0.05). *p* > 0.05 and *p* < 0.10 were considered a tendency. Fecal score was analyzed by Kruskal-Wallis test (p < 0.05).

The relative abundance of bacterial phyla and genera was compared using multiple linear regression analysis with covariate adjustment (MaAsLin2), considering *p* < 0.05. The analyses were conducted using Minitab 18 statistical software program (Minitab® Inc., United States) and MicrobiomeAnalyst 2.0 (28).

### 2.2 Experiment II: palatability assay

#### 2.2.1 Animals and facilities

This experiment was conducted in accordance with the previously described ethical approval (protocol n. 044/2023). Sixteen adult Beagle dogs (8 males and 8 females), with 1 year of age, neutered/spayed, and an average body weight of 10.47 ± 1.02 kg were used. The dogs had a mean BCS of 4.5 ± 0.51, assessed on a scale of 1 to 9, according to Laflamme (17). The health conditions and facilities were the same as described in Experiment I. The dogs were individually housed in kennels only during the palatability test (for about 30 min per day).

#### 2.2.2 Palatability test and experimental design

The palatability of the diets was assessed using first choice and intake ratio. The following diets were tested: 0% SCP vs. 4% SCP; 0% SCP vs. 8% SCP; and 4% SCP vs. 16% SCP. In each test, both diets were offered simultaneously. The amount of each diet offered in each bowl was 30% higher than the daily recommendations of the NRC (19) for the maintenance of adult dogs. Dogs were fed once daily in the morning during the palatability test. Once one of the diets was completely consumed, both bowls were withdrawn and the leftovers were quantified. The relative position of bowls was alternated on the second day of the experiment so that the animal was not conditioned to the feeding site. The first choice was defined by noting the first bowl that the animal approached. To determine the intake ratio, the amount of each diet offered, and the leftovers were quantified.

Each test was performed for two consecutive days according to a completely randomized design, totaling 32 repetitions per test.

#### 2.2.3 Calculations and statistical analysis

The intake ratio was calculated as:


$$\text{Intake ratio}=\frac{\text{intake of diet}\;(g)\;A\;\text{or}\;B}{\text{total consumed}\;(g)\;(A+B)\;}$$

Intake ratio data were analyzed by paired Student’s t-test (*p* < 0.05) and the first choice by McNemar’s test (p < 0.05).

### 2.3 Experiment III: digestibility of the ingredient

#### 2.3.1 Animals, facilities, diets, and experimental design

This experiment was conducted under the same ethical approval (protocol n. 044/2023). Twelve adult Beagle dogs (6 males and 6 females), 1 year of age, neutered/spayed, and with an average body weight of 10.53 ± 1.05 kg were used. The dogs had a mean BCS of 4.5 ± 0.55, according to Laflamme (17). The animals were kept in the same conditions described in Experiment I.

Two diets were used: control (0% SCP) and test, a diet containing 800 g/kg of the control formulation and 200 g/kg of SCP, according to the substitution method of Matterson et al. (29), with the aim of isolating the digestibility of the ingredient. Table 1 presents the ingredient composition of this diet. The manufacture of the test diet was the same as described in Experiment I, with SCP incorporated into the formulation prior to extrusion. The experiment followed a completely randomized design, totaling 6 repetitions per diet.

#### 2.3.2 Digestibility assay

The digestibility trial followed the total fecal collection method recommended by the AAFCO (20). For this, each 6 dogs were fed one of the diets (control or test) for 10 days, with 5 days of adaptation, followed by 5 days of total fecal collection. All the procedures and analysis for the digestibility assay were the same as described in Experiment I.

#### 2.3.3 Calculations

The ATTD of DM, CP, AEE and OM of the SCP were calculated according to the substitution method proposed by Matterson et al. (29). The following equations were applied:


$$\begin{array}{l} \text{ATTD of the}\;\mathrm{SCP}\;(\%)=\text{ATTD of control diet}\;(\%)+\\ \frac{\begin{array}{l} \text{ATTD of test diet}\;(\%)-\\ \text{ATTD of control diet}\;(\%) \end{array}}{(\begin{array}{l} g\;\text{of ingredient substitution}\\ \;\mathrm{on}\;a\;\mathrm{DM}\;\text{basis} \end{array})/1000} \end{array}$$

The digestible protein and fat of SCP were calculated as:


$$\begin{array}{l} \text{Digestible nutrient}\;(\%)=\\ \frac{\%\text{nutrient in}\;\mathrm{SCP}\;x\;\text{ATTD of the nutrient in}\;\mathrm{SCP}}{100} \end{array}$$

### 2.4 Results

#### 2.4.1 Experiment I: digestibility, fecal characteristics, fermentative metabolites, and microbiota

All dogs consumed all the food offered. No adverse effects to diets were observed, such as episodes of vomiting, diarrhea, or feed refusal.

The increasing dietary concentrations of SCP did not alter the DM intake and the ATTD of AEE (*p* > 0.05; Table 4). However, the ATTD of DM, OM, and GE and ME decreased linearly as the dietary inclusion of SCP increased (*p* < 0.05; Table 4). There was a tendency for a linear reduction in the ATTD of CP as the dietary inclusion of SCP increased (*p* = 0.051; Table 4).

**Table 4:** Means of dry matter intake (DMI), apparent total tract digestibility (ATTD, %), metabolizable energy (ME, kcal/kg), and fecal characteristics of dogs fed diets containing Single Cell Protein (SCP).

Item	SCP (%)					SEM^1^	P-L^2^	P-Q^3^	P^4^
	0	4	8	12	16				
DMI (g/dog/day)	251.8	240.1	249.9	228.2	229.9	4.60	0.087	0.901	-
ATTD (%)									
Dry matter	84.2	84.7	82.4	81.7	81.7	0.35	<0.001	0.778	-
Organic matter	86.6	86.8	85.3	84.2	84.3	0.30	<0.001	0.896	-
Crude protein	85.6	84.5	83.7	83.4	83.4	0.41	0.051	0.402	-
Ether extract	90.8	91.6	90.8	90.7	90.4	0.23	0.344	0.514	-
Gross energy	87.2	87.1	85.6	84.8	84.7	0.27	<0.001	0.739	-
ME (kcal/kg)	4320.8	4340.0	4252.3	4219.2	4209.2	14.10	<0.001	0.969	-
Fecal characteristics									
Dry matter (%)	34.4	32.6	31.6	30.4	30.9	0.48	0.005	0.220	-
Production^5^ (g/day)	115.9	113.8	135.7	138.7	135.8	4.46	0.038	0.569	-
Score^6^	3.6 (3.5–3.7)	3.7 (3.4–4.0)	3.7 (3.3–4.0)	3.6 (3.5–3.9)	3.7 (3.6–3.9)	-	-	-	0.652
pH	6.4	6.2	6.6	6.5	6.5	0.05	0.154	0.824	
Fermentative metabolites									
Ammonia (μmol/g)	0.3	0.2	0.2	0.2	0.2	0.01	0.276	0.222	

1 SEM, standard error of the mean.

2 P-L, probability for linear effect (*p* < 0.05).

3 P-Q probability for quadratic effect (*p* < 0.05).

4 Probability.

5 Production = g feces produced as-is/animal/day.

6 Score: Median (1°-3°quartiles) analyzed by Kruskall- Wallis (*p* < 0.05).

Regarding fecal characteristics, there was a linear reduction in DMf and a linear increase in the fecal production, as SCP dietary inclusion increased (*p* < 0.05; Table 4). There was no effect of SCP inclusion on fecal score and pH (*p* > 0.05; Table 4).

Regarding fecal fermentative metabolites, there was no effect of SCP inclusion on fecal concentrations of ammonia (*p* > 0.05). However, there was a quadratic effect in fecal concentrations of propionate, butyrate, total SCFA, and heptanoic acid with the dietary inclusion of SCP (*p* < 0.05; Table 5). On the other hand, there was a linear increase in fecal concentrations of isobutyrate, 4-methyl valerate, and total BCFA (*p* < 0.05; Table 5). There were no effects of SCP on other fermentative metabolites (*p >* 0.05; Table 5).

**Table 5:** Means of fecal concentrations of short-chain (SCFA), branched-chain (BCFA), and other volatiles fatty acids in dogs fed diets containing Single Cell Protein (SCP).

Item	SCP (%)					SEM^1^	P-L^2^	P-Q^3^
	0	4	8	12	16			
SCFA (mM/mol dry matter)								
Acetate	144.7	159.6	153.9	166.2	144.6	4.77	0.818	0.091
Propionate	45.6	60.8	55.7	63.8	57.0	2.13	0.039	0.027
Butyrate	17.4	22.3	23.1	23.6	22.1	0.91	0.071	0.049
Total SCFA	207.7	242.7	232.7	253.6	223.7	6.96	0.274	0.027
Valerate	6.5	6.6	7.0	6.6	6.7	0.13	0.722	0.500
BCFA and volatiles (mM/mol dry matter)								
Isovalerate	5.2	5.3	5.6	5.5	5.5	0.08	0.182	0.318
Isobutyrate	5.7	6.0	6.2	6.5	6.2	0.09	0.037	0.164
4-methyl valerate	0.6	0.6	0.6	0.8	0.7	0.02	0.013	0.497
Total BCFA	11.5	11.7	12.4	12.7	12.4	0.18	0.048	0.193
Hexanoic	1.6	1.0	1.1	1.5	1.2	0.08	0.654	0.152
Heptanoic	4.5	4.8	5.2	5.2	5.0	0.07	0.014	0.010

1 SEM, Standard error of the mean.

2 P-L, probability for linear effect (*p* < 0.05).

3 P-Q, probability for quadratic effect (*p* < 0.05).

Regarding fecal microbiota analyses, no differences were observed at the phylum level (*p* > 0.05). Nevertheless, the core bacterial phyla observed in all groups fed 0, 4, and 8% SCP were Bacteroidota, Bacillota (previously Firmicutes), Fusobacteriota, Proteobacteria, and Actinobacteriota.

**Figure 1:**
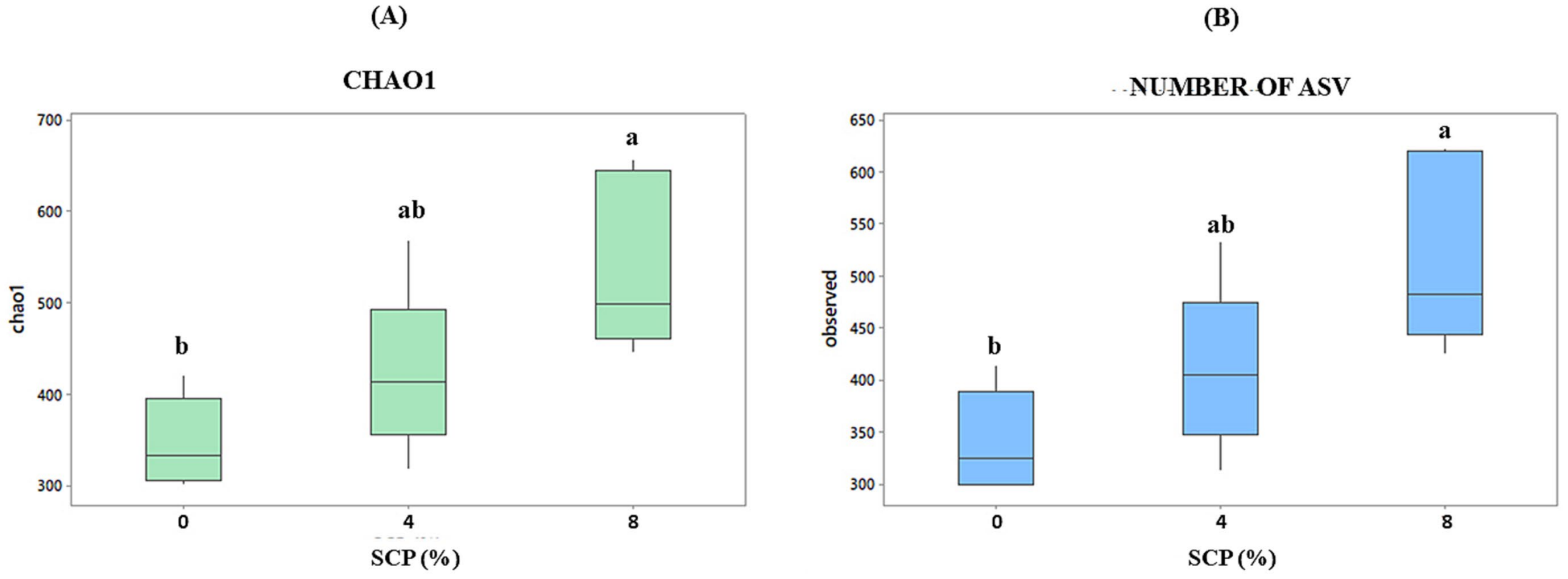
Alpha-diversity indexes of fecal microbiota of dogs fed diets containing 0, 4, and 8% single-cell protein (SCP): Chao1 index **(A)** and number of amplicon sequence variants (ASVs) **(B)**. *p* < 0.05 by ANOVA F test. ^ab^Means with different letters were statistically different according to the Tukey test (*p* < 0.05).

There was no effect of SCP in the Shannon index (0%: 3.78; 4%: 3.98; and 8%: 4.15; *p* > 0.05). However, an increase in the Chao1 index and the number of ASVs was observed in the feces of dogs fed 8% SCP when compared to the control group (*p* < 0.05, Figure 1).

**Figure 2:**
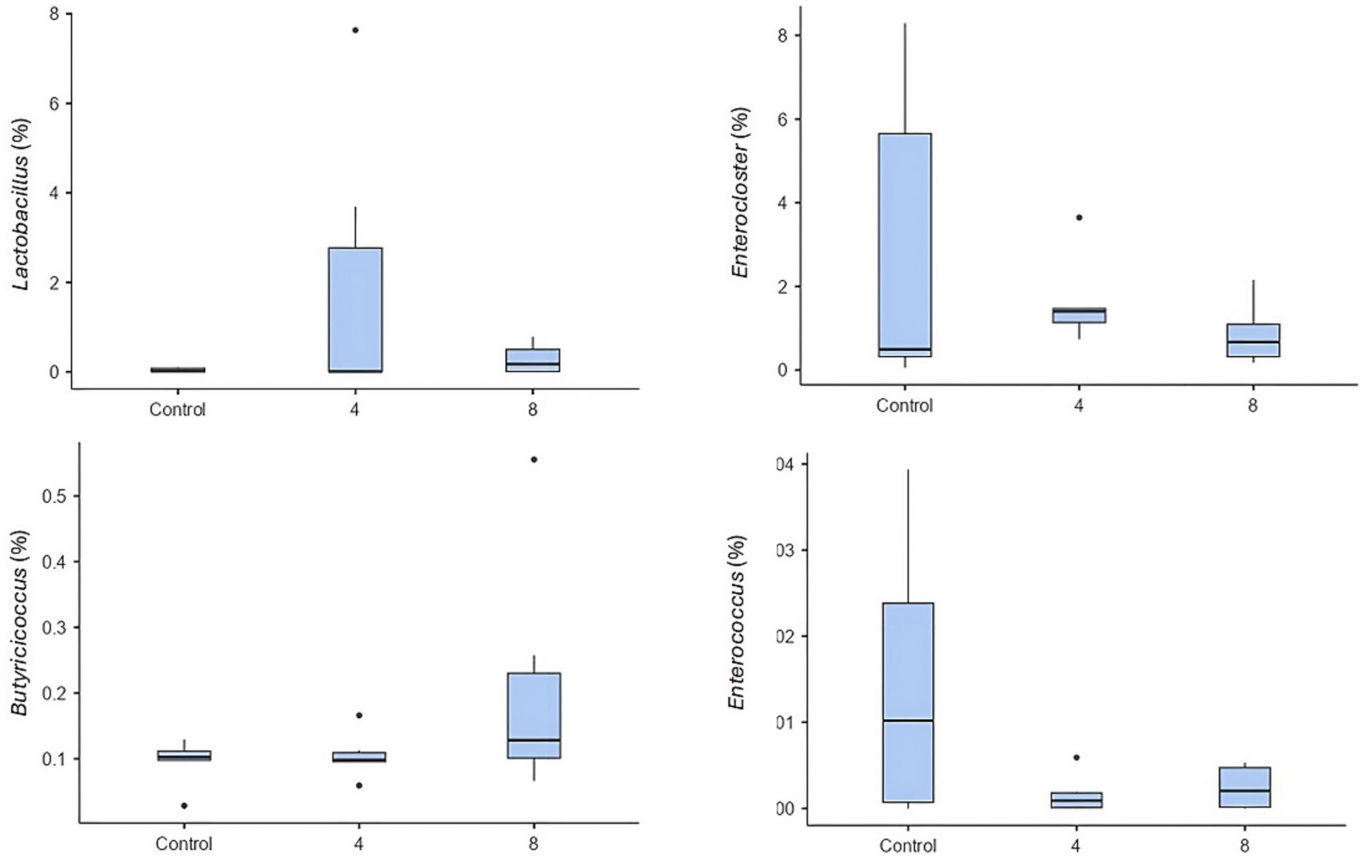
Relative abundance (%) of the genera *Butyricicoccus*, *Lactobacillus*, *Enterocloster*, and *Enterococcus* in the feces of dogs fed diets containing 0, 4, and 8% of single cell protein (SCP). *p* < 0.05 by multiple linear regression test with adjustment for covariates.

The main bacterial genera increased in the feces of dogs consuming the 4% SCP diet compared to the control diet were: *Helicobacter* (*p* < 0.05; Table 6), *Lactobacillus* (*p* < 0.05; Table 6; Figure 2), *Limosilactobacillus,* and *Duodenibacillus* (*p* < 0.05; Table 6). In contrast, the genera *Enterococcus* (*p* < 0.05; Table 6; Figure 2), *Merdicola,* and *Faecalibaculum* were reduced in the animals fed the 4% SCP diet compared to the control group (*p* < 0.05; Table 6).

**Table 6:** Log_2_ fold change (FC) of the main bacterial genera in the feces of dogs fed 4% of single cell protein (SCP) compared to the control group (0% SCP).

Item	0 vs. 4% SCP	
	Log_2_ FC	P^1^
*Helicobacter*	2.02	0.001
*Lactobacillus*	3.53	0.002
*Merdicola*	−4.56	0.006
*Enterococcus*	−2.07	0.014
*Faecalibaculum*	−4.68	0.020
*Limosilactobacillus*	2.16	0.031
*Duodenibacillus*	0.86	0.044

1 Probability.

Negative Log_2_ FC values represent a decrease, while positive values represent an increase, compared to the control.

An increase in the *Lactobacillus* genus was also observed in the animals fed the 8% diet compared to the control group (*p* < 0.05; Table 7; Figure 2). In addition, these animals presented an increase in the genera *Butyricicoccus* (*p* < 0.05; Table 7; Figure 2), *Emergencia*, and *Parabacteroides* (*p* < 0.05; Table 7), as well as a reduction in the genera *Merdicola* (*p* < 0.05; Table 7), *Enterococcus*, and *Enterocloster* (*p* < 0.05; Table 7; Figure 2).

**Table 7:** Log_2_ fold change (FC) of the main bacterial genera in the feces of dogs fed 8% of single cell protein (SCP) compared to the control group (0% SCP).

Item	0 vs. 8% SCP	
	Log_2_ FC	P^1^
*Emergencia*	2.86	0.006
*Merdicola*	−5.25	0.005
*Enterococcus*	−1.78	0.025
*Butyricicoccus*	0.778	0.032
*Enterocloster*	−1.29	0.046
*Lactobacillus*	2.17	0.038
*Parabacteroides*	1.42	0.048

1 Probability.

Negative Log_2_ FC values represent a decrease, while positive values represent an increase, compared to the control.

#### 2.4.2 Experiment II: palatability assay

There was no difference in the first choice and intake ratio among the diets (*p* > 0.05; Table 8). The intake ratio ranged from 0.43 to 0.57, with no consistent preference for any treatment (Table 8).

**Table 8:** Number of first choices (N) and intake ratio (IR) of the 0, 4, 8, and 16% single cell protein diets.

A x B	NA	NB	IRA	IRB	P-N^1^	P-IR^2^
0% vs. 4%	19	13	0.46	0.54	0.377	0.498
0% vs. 8%	15	17	0.57	0.43	0.860	0.271
4% vs. 16%	15	17	0.45	0.55	0.860	0.415

1 P-N, probability by McNemar test for first choice.

2 P-IR, probability by paired Student’s t-test for intake ratio.

IR: [g of feed intake of A or B/g of feed intake (A + B)].

#### 2.4.3 Experiment III: digestibility of the ingredient

The dogs normally consumed the test diet with 20% SCP used to calculate the digestibility of the ingredient (mean = 255 g DM/animal/day). They also presented a DMf and fecal score of 29.7% and 3.9, respectively, and fecal production of 165 g (as-is/animal/day). Table 9 presents the ATTD and digestible nutrients of the SCP evaluated.

**Table 9:** Means ± standard error of apparent total tract digestibility (ATTD, %) and metabolizable energy (ME, kcal/kg) and digestible nutrients of single cell protein (SCP).

Item	SCP
ATTD (%)	
Dry matter	64.3 ± 0.56
Organic matter	61.4 ± 0.34
Crude protein	83.9 ± 1.26
Ether extract	78.3 ± 1.96
Gross energy	67.9 ± 1.29
ME (kcal/kg)	3843.3 ± 66.1
Digestible nutrients (%, DM)	
Digestible protein	57.5
Digestible ether extract	2.8

## 3 Discussion

Evaluating an ingredient for pet food applications requires a comprehensive understanding of its nutritional value, digestibility, and functional characteristics, which are closely related to its chemical composition. According to the chemical analysis, SCP presented greater CP concentration (65.7%) in comparison to plant sources (E.g. soybean meal—50% CP) (30) and microbial sources such as sugarcane and brewer’s yeast (40–50% CP) (31). In addition, this SCP exhibits a favorable amino acid profile, with concentrations that align with FEDIAF nutritional recommendations for dogs (18). Notably, its methionine content (0.89% DM) is relatively high, an uncommon characteristic among fungal proteins, which typically contain low concentrations of sulfur amino acids (8).

Regarding other nutritional fractions, the evaluated SCP has an ash content (7%) that is relatively similar to conventional soybean meal (6.4%) and lower than that observed in animal protein sources, such as isolated porcine protein (10%) and poultry by-product meal (14–18%) (30, 32). This nutritional profile offers potential advantages over conventional ingredients of plant or animal origin.

Despite its promising nutritional profile, a possible limitation to the use of this SCP in dog food is its high total dietary fiber (TDF) content (22.9% in DM basis) of which 1.4% corresponds to soluble and 21.5% to insoluble fiber. This fiber profile may reduce nutrient digestibility and negatively impact fecal consistency depending on SCP dietary concentration (33). This potential negative impact of SCP on diet digestibility was observed in the present study, especially on DM, OM, and GE and on ME.

The observed reduction in diet digestibility can be attributed mainly to two factors. The first is related to the amount and type of fiber present in the ingredient. Soluble fiber can increase the viscosity of intestinal contents, making it more difficult for digestive enzymes to interact with their substrates (34). On the other hand, a high proportion of insoluble fiber can compromise nutrients digestibility by accelerating gastrointestinal transit time, reducing the exposure time of nutrients to absorption sites (33). The second factor involves the composition of the fungal cell wall, characterized by the presence of structural polysaccharides (9).

The cell wall of *Paecilomyces* var*iotii* is mainly composed of *α*-(1,3)-glucan (33.2–39.1%) and a *β*-glucan-chitin complex (42.7–47.3%), as well as soluble fractions (2.8–4.7% of the cell wall) containing β-1,3/1,6-glucan along with mannose, glucose, and galactose, including manopyranose and galactofuranose residues (9). Among these constituents, chitin, a structural polysaccharide with high resistance to enzymatic digestion, is notable. In dogs, the limited expression of acid chitinase reduces the ability to degrade this molecule (35), which may have contributed to the lower digestibility observed with the increased dietary inclusion of SCP in this study. Similar results were observed in studies with Atlantic salmon, in which the progressive inclusion of the same SCP (*P.* var*iotti*, 0, 4, and 8%) as a protein substitute resulted in a linear reduction in the ATTD of CP and GE (16).

Despite the reduction in macronutrient digestibility, the ATTD of DM of experimental diets remained consistently high, exceeding 80%. These results are consistent with the isolated digestibility values of the ingredient, which had an ATTD of DM of 64.3% and an average ATTD of 83.9% for CP. Given the ingredient’s high TDF content, these values are quite satisfactory. Protein digestibility values above 80% are normally observed in high-quality animal and vegetable protein sources in dogs (32, 36).

It is important to highlight that ingredients with high fiber content do not usually present high ATTD of CP. For example, yeast extract, another source of microbial protein with 38–44% CP and high structural fiber content, may present ATTD of CP of 55% when estimated by the substitution method in dogs (37). This difference can be explained by structural variations in the cell wall: yeasts have a thick, rigid cell wall that can limit enzymatic access and protein utilization (3), while filamentous fungi, such as *P. variotii*, have a less encapsulated biomass structure, which probably allows for better enzymatic penetration (38).

Among the fecal characteristics evaluated, changes in DMf and fecal output observed in dogs fed increasing dietary inclusion of SCP can be explained by the physicochemical properties of the ingredient. Although no previous studies in dogs or other monogastric species have reported fecal score or moisture with fungal SCP inclusion, these changes align with the soluble fiber content in the SCP, which is known to retain water in the colon (39). Despite these effects, fecal scores remained within the optimal range (3.6–3.7), indicating well-formed and consistent stools, an important consideration from the pet owner’s perspective.

As mentioned above, the cell wall of *Paecilomyces variotii* contains a soluble fraction with around 11% *β*-1,3/1,6-glucans (9, 40). Although no studies have investigated the effects of this SCP in dogs, there is evidence of the potential functional effects of β-glucans extracted from the fungus *Hericium erinaceus* and the algae *Euglena gracilis*, on the microbiota and immune system of dogs (12, 41). The modulation of the gut microbiome can occur through two non-exclusive mechanisms: (I) the prebiotic effect of SCP cell wall components, such as mannans and β-glucans, which serve as a substrate for fermentation by colonic bacteria, resulting in the production of SCFA (42), and (II) the immunomodulatory effect of β-1,3/1,6-glucans, which interact with the Dectin-1 receptor, promoting the reduction of inflammation and, as a consequence, the indirect modulation of the gut microbiota (43).

Corroborating the first proposed mechanism, SCP inclusion was associated with greater fecal concentrations of propionate, butyrate, and total SCFAs in the present study. Elevated concentrations of these metabolites are indicative of eubiosis in dogs (44, 45) due to their beneficial effects on the intestinal environment, including reduction of luminal pH, inhibition of the growth of potentially pathogenic bacteria, and energy supply to colonocytes (46). In addition, propionate and butyrate have well-established anti-inflammatory properties, acting in the negative modulation of receptors such as Toll-like receptor 4 and in the inhibition of the NF-κB signaling pathway (47–49), which results in the suppression of pro-inflammatory cytokine production (50, 51).

*In vitro* fermentation studies have demonstrated that both the mycoprotein derived from the filamentous fungus *Fusarium venenatum* and its isolated fiber fraction are fermentable, resulting in significant production of SCFA, especially propionate and butyrate, which supports the functional potential of fungal fibers in modulating the fecal microbiota (42). In studies with fish fed diets containing *P.* var*iotii*, although SCFAs or microbial composition were not directly evaluated, an increase in the area occupied by goblet cells in the intestinal mucosa was observed, suggesting increased mucus secretion and, consequently, reinforcement of the intestinal physical barrier. In addition, all diets containing *P. variotii* (4, 8, and 16%) promoted the expression of cytokines and transcription factors associated with anti-inflammatory, antioxidant, and regulatory responses (16).

Despite the lack of differences in fecal ammonia concentrations, an important end-product of protein fermentation, our results indicate an increase in fecal concentrations of isobutyrate, 4-methyl valerate, total BCFAs, and heptanoic acid in response to SCP dietary inclusion. These metabolites result from the fermentation of undigested proteins in the colon and have been associated with potential mucosal toxicity (52). Although high protein digestibility was observed, the CP values, based on total nitrogen, may have been overestimated due to the presence of non-protein nitrogen, particularly nucleic acids (7–10%), which are commonly present in fungal biomass (8, 53). Nevertheless, it is important to note that there are currently no reference values available in the literature to define fecal BCFA concentrations considered harmful to dogs.

In this context, the analysis of the fecal microbiota revealed that the inclusion of SCP at 4 and 8% was associated with changes in fecal microbiota and fermentative metabolites in dogs. This modulation was evidenced by higher Chao1 index values and a greater number of ASVs in the feces of dogs fed 8% SCP compared to the control group. This increase in microbial richness may be indicative of a more balanced environment, as it is commonly associated with eubiosis in dogs (44).

Regarding fecal microbiota composition, SCP inclusion, particularly at 4%, was associated with a greater relative abundance of *Limosilactobacillus*, *Duodenibacillus*, and *Lactobacillus* (also increased in the 8% SCP diet group)*. Limosilactobacillus* and *Lactobacillus* are well-known lactic acid producers, contributing to the acidification of the intestinal lumen and the consequent reduction of bacteria with pathogenic potential. In addition, some species of these genera are often used as probiotics for dogs (54–56). Similar changes in fecal microbiota composition of broilers were reported following supplementation with polysaccharides from *Hericium caput-medusae*, suggesting a consistent prebiotic effect of fungal bioactives across species, likely due to the presence of fungal *β*-glucans and chitin, which are selectively fermented by saccharolytic bacteria in the colon, such as *Lactobacillus* (57). Moreover, laying hens fed SCP from microbial origin, combined with multistrain probiotics, also exhibited increased *Lactobacillus* abundance and were associated with improvements in intestinal health markers (58). It is important to note that these findings derive from studies using supplementation with fungal components or microbial protein, rather than dietary replacement of traditional protein sources. *Duodenibacillus* is a bacterial genus recently identified in the human intestine (59) and associated with patients who have fully recovered from cancer (60). This genus is taxonomically similar to the genus *Sutterella*, which is related to eubiosis in dogs (61).

Another genus that also increased in response to the inclusion of 8% SCP was *Butyricicoccus*. This result aligns with the quadratic effect observed in fecal concentrations of butyrate in this study, since this genus is recognized as a butyrate producer (62, 63). In addition, *Butyricicoccus* has been associated with the high consumption of fibers (64), which is consistent with the chemical profile of the SCP evaluated. Surprisingly, the genus *Faecalibaculum* was reduced in the feces of dogs fed 4% SCP. This genus is also correlated with SCFA production (65, 66). However, as previously discussed, SCFA concentrations demonstrated a quadratic response to SCP inclusion, suggesting the overall fermentative activity was maintained. This finding may be explained by the functional redundancy of the gut microbiota (67), in which different bacteria can perform similar metabolic functions.

In contrast, some bacterial genera exhibited a reduction in dogs fed diets containing SCP compared to the control diet, as observed for the genus *Enterocloster* in the diet with 8% SCP and for the genus *Enterococcus* in diets with 4 and 8% SCP. According to the literature, both genera have been associated with pathological conditions. *Enterocloster* is related to the development of colorectal cancer in humans (68). Similarly, although the genus *Enterococcus* includes species with probiotic potential, such as *Enterococcus faecium*, its increase in feces has been correlated with greater antimicrobial resistance, in addition to being present in dogs with hyperadrenocorticism and in opportunistic infections of the gastrointestinal and urinary tract (69, 70).

Finally, a reduction in the fecal abundance of the genus *Merdicola* was observed in dogs fed both diets containing 4 and 8% SCP. This taxon, which is still undercharacterized, belongs to the phylum Bacillota (previously Firmicutes) and the class Clostridia (71). In dogs, some genera belonging to Bacillota, such as *Lactobacillus*, have a positive correlation with the production of SCFA (72). On the other hand, this phylum also includes bacterias with pathogenic potential, such as some *Clostridium* species. Thus, given the lack of data on *Merdicola*, it is not yet possible to establish a precise interpretation of its reduction associated with the inclusion of SCP in the diet.

In addition to its effects on the fecal microbiota, another important aspect of incorporating new ingredients is their acceptance by the animal. In this context, diet palatability is one of the most important factors considered by pet food companies. In the present study, we observed that the dietary inclusion of up to 16% SCP did not interfere with the palatability. Despite this, studies with SCP, in general, demonstrate that the response may vary according to the level of inclusion and the animal species (73, 74). Specifically, although studies with *P. variotii* did not directly evaluate palatability, inclusion levels of up to 15–20% in poultry, pigs, and fish did not impair voluntary consumption, suggesting good acceptance of the ingredient by these species (13–16, 75).

To the best of our knowledge, this is the first study to evaluate the nutritional and functional aspects of *P. variotii* as an unconventional protein source for dogs. Despite these potential negative effects on nutrient digestibility, it is important to note that the high fiber content of this ingredient may be advantageous in specific formulations, such as weight management diets, in which reduced energy density is desirable. In addition, considering the high content of *β*-glucans present in SCP and its functional potential, evidenced by the modulation of the microbiota and intestinal environment observed in this study, and considering that β-glucans can modulate the immune system of dogs and reduce inflammation (41, 76, 77), it would be interesting to investigate how this ingredient can impact the canine immune system in further studies. However, some limitations should be considered, including the relatively small number of animals, especially for palatability test, the evaluation of microbiota only in selected dietary treatments (0, 4, and 8% SCP), and the use of healthy adult Beagle dogs over a relative short feeding period. Therefore, these findings should be interpreted with caution when extrapolated to other breeds, life stages, or long-term feeding conditions.

## 4 Conclusion

The dietary inclusion of up to 16% SCP reduced DMf and the digestibility of DM, OM, and GE, and the ME of the diet while increasing both fecal output and fecal BCFA concentrations. Despite these changes, there was no effect on fecal score and CP digestibility. Additionally, the inclusion of SCP may contribute to beneficial modulation of the intestinal environment, evidenced by increased production of butyrate, propionate, and total SCFAs, as well as an increase in genera associated with eubiosis, such as *Lactobacillus* and *Butyricicoccus*, and a reduction in potentially pathogenic genera, such as *Enterococcus* and *Enterocloster*. Furthermore, the inclusion of SCP did not affect diet palatability in dogs. These findings highlight the potential of SCP as an alternative protein source with functional properties in dog nutrition. However, further studies are warranted to confirm these results in larger populations and under different feeding conditions.

## Data Availability

The original contributions presented in the study are publicly available. This data can be found here: https://doi.org/10.5281/zenodo.20395872.
